# National Dissemination of Multiple Evidence-Based Disease Prevention Programs: Reach to Vulnerable Older Adults

**DOI:** 10.3389/fpubh.2014.00156

**Published:** 2015-04-27

**Authors:** Samuel D. Towne, Matthew Lee Smith, SangNam Ahn, Mary Altpeter, Basia Belza, Kristie Patton Kulinski, Marcia G. Ory

**Affiliations:** ^1^Department of Health Promotion and Community Health Sciences, Texas A&M Health Science Center School of Public Health, College Station, TX, USA; ^2^Department of Health Promotion and Behavior, College of Public Health, The University of Georgia, Athens, GA, USA; ^3^Division of Health Systems Management and Policy, School of Public Health, The University of Memphis, Memphis, TN, USA; ^4^University of North Carolina at Chapel Hill, Chapel Hill, NC, USA; ^5^Biobehavioral Nursing and Health Systems, School of Nursing, University of Washington, Seattle, WA, USA; ^6^National Council on Aging, Washington, DC, USA

**Keywords:** evidence-based programs, community intervention, minority adults, older adults, aging health

## Abstract

Older adults, who are racial/ethnic minorities, report multiple chronic conditions, reside in medically underserved rural areas, or have low incomes carry a high burden of chronic illness but traditionally lack access to disease prevention programs. The Chronic Disease Self-Management Program (CDSMP), A Matter of Balance/Volunteer Lay Leader (AMOB/VLL), and EnhanceFitness (EF) are widely disseminated evidence-based programs (EBP), but the extent to which they are simultaneously delivered in communities to reach vulnerable populations has not been documented. We conducted cross-sectional analyses of three EBP disseminated within 27 states throughout the United States (US) (2006–2009) as part of the Administration on Aging (AoA) Evidence-Based Disease and Disability Prevention Initiative, which received co-funding from the Atlantic Philanthropies. This study measures the extent to which CDSMP, AMOB/VLL, and EF reached vulnerable older adults. It also examines characteristics of communities offering one of these programs relative to those simultaneously offering two or all three programs. Minority/ethnic participants represented 38% for CDSMP, 26% for AMOB/VLL, and 43% for EF. Rural participation was 18% for CDSMP, 17% for AMOB/VLL, and 25% for EF. Those with comorbidities included 63.2% for CDSMP, 58.7% for AMOB/VLL, and 63.6% for EF while approximately one-quarter of participants had incomes under $15,000 for all programs. Rural areas and health professional shortage areas (HPSA) tended to deliver fewer EBP relative to urban areas and non-HPSA. These EBP attract diverse older adult participants. Findings highlight the capability of communities to serve potentially vulnerable older adults by offering multiple EBP. Because each program addresses unique issues facing this older population, further research is needed to better understand how communities can introduce, embed, and sustain multiple EBP to ensure widespread access and utilization, especially to traditionally underserved subgroups.

## Introduction

The aging of the US population has far reaching effects on the American health care system (1). Chronic disease is becoming endemic among older Americans (2). National statistics indicate most adults aged 65 and older have at least one chronic condition (91%), while nearly three-quarters have two or more chronic conditions (2). Additionally, age-related geriatric conditions are prevalent in this population and have stark public health consequences. Each year, falling affects approximately one-third of older adults in the US (3) contributing to death and serious injuries and costing billions of dollars in healthcare expenses annually (estimated to reach $30 billion by 2020) (4–8). In addition, high blood pressure, high cholesterol, heart disease, arthritis, and diabetes are common among older adults (9), and in many cases comorbidities are also present (10).

Self-management is seen as a critical component of clinical- and community-based health care (11, 12). Although self-management strategies are widely promoted (13), individuals with multiple chronic conditions experience barriers to successful self-care (14). Given that older adults have different chronic diseases, varying comorbidity combinations, and are at differing stages of disease progression, there is need for multiple intervention approaches in any given community.

In concert with public health officials and policy makers’ interests to identify effective ways to lessen the impact of chronic disease and other complications among the aging population [e.g., Healthy People 2020 (15)], evidence-based programs (EBP) for older adults have emerged and proliferated in the US (16–20). In recent years, multiple EBP have been disseminated through the US aging services network to address different healthcare concerns experienced by older adults (21). However, there is no “one size fits all” EBP, which highlights the need for communities to introduce multiple programs to meet the various needs of a diverse aging population.

While it is assumed that distinct EBP attract specific types of participants (17, 18) and certain types of participants are more likely to attend EBP at particular types of delivery sites (16), the extent to which EBP attract and retain potentially vulnerable older adults is not fully understood. Older adults deemed vulnerable can include those with comorbid conditions (22), in advanced age, and of racial/ethnic minority status (23–25). Vulnerability can also be defined as older adults residing in areas with limited resources, which include rural areas (26–28), those with limited health care providers, or those with high poverty rates compared to most other areas (29, 30). As such, the purpose of this study was twofold: (1) to measure the extent to which three widely disseminated EBP reached vulnerable older adults and (2) to assess the extent to which delivery areas offered multiple EBP.

## Materials and Methods

### Selected evidence-based programs

For the purposes of this study, three EBP for older adults were examined. The programs included in this study include: Stanford University’s Chronic Disease Self-Management Program (CDSMP), A Matter of Balance/Volunteer Lay Leader (AMOB/VLL), and EnhanceFitness (EF). Each program was selected because of its national dissemination spanning multiple states and well-documented effectiveness for improving health outcomes in community settings.

These EBP have demonstrated their effectiveness in improving health among older adults. CDSMP targets adults with multiple chronic conditions (e.g., teaching self-management skills) and has been shown to be effective at delaying the onset of illness and helping participants improve the management of multiple chronic diseases while reducing hospitalizations (31–34). AMOB/VLL targets older adults, especially those at risk of falling (35) and has been shown to reduce the fear of falling, improve long-term social functioning, and improve long- and short-term mobility in older adults (17, 36–38). EF is a group exercise program (39) that has been shown to improve upper and lower body muscle strength, depression (40), and lower healthcare costs (41).

### Data analyses

We conducted a cross-sectional analysis of three EBP. Participant data and information about program delivery locations were drawn from the National Council on Aging’s database of 24 states implementing EBP from 2006 to 2009 as part of the Administration on Aging (AoA) Evidence-Based Disease and Disability Prevention Initiative and 3 states funded by the Atlantic Philanthropies (16). Only data collected between 2006 and 2009 from these initiatives were included in these analyses. These data were linked with the 2013 Area Health Resource File (AHRF) to identify Primary Care Health Professional Shortage Areas (HPSA) and Urban Influence Codes (UIC) (42). HPSA is classified into full-HPSA, partial (only a portion of the county was classified as a HPSA), and a non-HPSA. A HPSA is classified based on geographic area and population size (e.g., primary care physician ratio of less than 3,500 to 1) (43). Rural areas were defined as having a UIC of ≥3 versus urban/metropolitan defined as having a UIC of 1–2. UICs take into consideration the population size and, for rural areas, the relative proximity to metropolitan or micropolitan areas (44). We used ArcGIS version 10.2 for all mapping of data presented in the figures (45). Chi-square tests were used to compare categorical study variables and independent sample *t*-tests were used to assess differences in continuous variables. We used SAS version 9.4 for all statistical analyses (46).

### Variables

#### Vulnerability

Vulnerable adults are the focus of our analysis. Acknowledging that vulnerability can be defined in numerous ways, the operational definition of vulnerability used in this study includes participants meeting one or more of the following criteria: being in advanced age (i.e., age 75 and older), having low income (i.e., self-reporting an annual household income <$15,000), being in a racial/ethnic minority (non-White), having one or more chronic conditions, living in a HPSA (47, 48), living in an area with poverty rates above the median (i.e., based on the percent Federal Poverty Rates in 2008 (14.1%) at the county level according to the 2013 AHRF), or living in a rural area (i.e., counties with UIC ≥3) (49). Only those individuals with one or more chronic conditions were included in our analyses.

#### Covariates

Sex of the participants who attended the EBP was reported. Income was categorical; however, a missing category for income was included in analyses, as we did not assume this was missing at random.

### Handling missing data

As described elsewhere (50), the AoA initiative required only a few participant level variables be collected, including age, sex, living alone status, race/ethnicity, and ZIP Code. Even this limited number of variables was not collected routinely by all state grantees; however, some states chose to routinely collect information related to chronic conditions and income. Missingness (i.e., missing data) was addressed independently according to the analysis performed and variables included. Independently (i.e., only considering each variable’s missingness exclusive of other missing variables), our sample size (*n* = 48,413) was gradually reduced when removing missing observations for race (*n* = 37,661), sex (*n* = 39,488), county Federal Information Processing Standard (FIPS) (*n* = 36,599), age (*n* = 35,248), the number of chronic conditions (*n* = 22,007), and income (*n* = 22,956). Dependently, when collectively removing observations for race, sex, county FIPS, and age, our sample size used in univariate and bivariate analysis was 30,185 observations.

## Results

### Reach into vulnerable populations

Table 1 presents the distribution of participant characteristics in the aggregate and by program type. Of the 30,185 participants enrolled in one of three EBP in this study, the majority participated in CDSMP (*n* = 16,612), followed by AMOB/VLL (*n* = 8,391), and EF (*n* = 5,182). On average, participants were aged 72.09 (±12.21) with 46.8% aged 75 and older. The majority of participants were female (80.8%), white (64.7%), and non-Hispanic (87.8%). The mean number of self-reported chronic conditions was 2 (±1.00). Approximately 24% of participants reported household incomes less than $15,000 per year, and 17.6% resided in rural areas.

**Table 1:** **Distribution of participant characteristics by program**.

	CDSMP (*n* = 16,612)		AMOB/VLL (*n* = 8,391)		EF (*n* = 5,182)		Total (*n* = 30,185)	
	*n*	%	*n*	%	*n*	%	*n*	%
**Age group**								
<50	1,323^a^^,^^b^	8.0	55^b^^,^^c^	0.7	135^a^^,^^c^	2.6	1,513	5.0
50–64	3,635^a^^,^^b^	21.9	656^b^^,^^c^	7.8	1,043^a^^,^^c^	20.1	5,334	17.7
65–74	5,151^a^^,^^b^	31.0	2,120^b^^,^^c^	25.3	1,933^a^^,^^c^	37.3	9,204	30.5
75 and older	6,503^a^^,^^b^	39.2	5,560^b^^,^^c^	66.3	2,071^a^^,^^c^	34.0	14,134	46.8
Age (mean)	69.6* (SD = 13.2)	77.5* (SD = 9.1)	71.4* (SD = 10.5)	72.1 (SD = 12.2)	Age (mean)	69.6* (SD = 13.2)	77.5* (SD = 9.1)	71.4* (SD = 10.5)
**Race/ethnicity**								
White	10,250	61.7	6,270	74.7	3,010	58.1	19,530	64.7
Black or African American	2,136	12.9	581	6.9	987	19.1	3704	12.3
American Indian/Alaska Native	147	0.9	221	2.6	180	3.5	548	1.8
Asian	882	5.3	151	1.8	265	5.1	1,298	4.3
Other	764	4.6	199	2.4	146	2.8	1,109	3.7
Hispanic	2,433	14.7	969	11.6	594	11.5	3,996	13.2
**Sex**								
Male	3,648	22.0	1,393	16.6	756	14.6	5,797	19.2
Female	12,964	78.0	6,998	83.4	4,426	85.4	24,388	80.8
**Number of chronic conditions**								
1	4,185	36.6	806	40.9	1,120	37.9	6,111	37.3
2	3,828	33.5	733	37.2	1,048	35.5	5,609	34.3
3	2,379	20.8	332	16.9	544	18.4	3,255	19.9
4	835	7.3	85	4.3	209	7.1	1,129	6.9
5 +	217	1.9	14	0.7	33	1.1	264	1.6
Average	2.04* (SD = 1.0)		1.87* (SD = 0.9)		1.98* (SD = 2.0)		2.01 (SD = 1.0)	
**Income**								
Missing	3,292	47.8	1,498	40.2	1,917	39.8	6,707	43.5
Less than $15,000	1,692	24.6	975	26.2	1,059	22.0	3,726	24.1
$15,000–24,999	820	11.9	479	12.9	742	15.4	2,041	13.2
$25,000–49,999	694	10.07	465	12.48	715	14.84	1,874	12.14
$50,000–75,000	251	3.64	199	5.34	254	5.27	704	4.56
More than $75,000	143	2.07	109	2.93	132	2.74	384	2.49
**Rurality**								
Rural	2,675	16.10	1,189	14.17	1,437	27.73	5,301	17.56
Urban	13,937	83.90	7,202	85.83	3,745	72.27	24,884	82.44

*Significantly (*p* < 0.05) different by program for select comparisons (i.e., age and the number of chronic conditions).

*^a^Significantly different CDSMP versus EF, within age group*.

*^b^Significantly different CDSMP versus AMOB/VLL, within age group*.

*^c^Significantly different AMOB/VLL versus EF, within age group*.

The average age of participants varied significantly (*p* < 0.05) across program types (i.e., 77.49 for AMOB/VLL, 71.39 for EF, 69.58 for CDSMP) with AMOB/VLL attracting the oldest participants. AMOB/VLL had the highest proportion of participants aged 75 years and older (66.3%) compared to 39.2% for CDSMP and 40.0% for EF. Those with comorbid conditions (i.e., 2 or more chronic conditions) represented 63.4% for CDSMP, 59.1% for AMOB, and 62.1% for EF. The average number of chronic diseases was significantly (*p* < 0.05) different for all comparisons across programs except CDSMP versus EF; CDSMP attracted participants with the most chronic conditions. CDSMP also attracted the largest proportion of Hispanic participants (14.7%). Those residing in rural areas represented 16.0% for CDSMP, 14.2% for AMOB/VLL, and 27.7% for EF. Those reporting incomes less than $15,000 per year were 24.6% CDSMP, 26.2% AMOB/VLL, and 22% EF.

**Figure 1:**
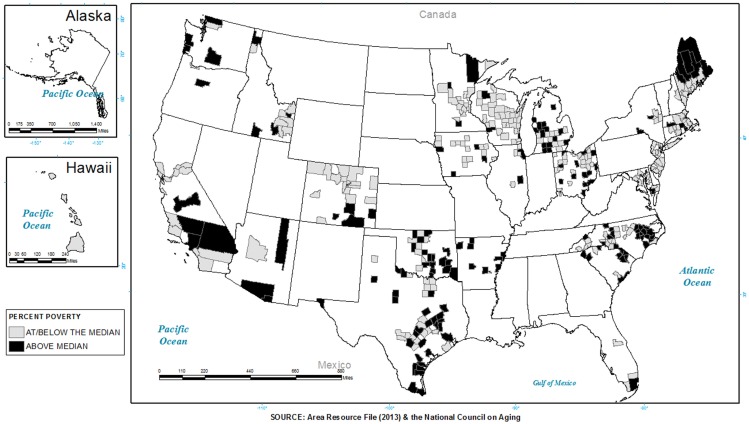
**The distribution of areas with a higher poverty rate than the median and a presence of evidence-based programs in 2006–2009**.

To graphically illustrate the extent to which programs were being delivered in areas classified as vulnerable by poverty rate or health access, a series of three maps highlighting participating states were constructed. Figure 1 shows where programs were delivered in areas with higher poverty rates than the 2008 median rate. States without shading include those states that were not included in the initiative. Gray shading represents where programs (i.e., CDSMP, AMOB/VLL, EF) were offered in areas equal to or below the 2008 median poverty rate. Black shading represents where programs were offered in areas *higher* than the 2008 median poverty rate. As seen, approximately 49.6% of the participants attended programs in areas with higher poverty rates. A greater proportion of participants in areas with higher poverty rates were served by EF at 58.4%, compared to 52.2% by AMOB/VLL and 45.5% by CDSMP. As can be seen, programs were delivered in high need areas, but the extent varied by state. For example, larger portions of California and North Carolina and smaller proportions of Oklahoma, Maine, and Washington delivered programs in areas with higher poverty.

**Figure 2:**
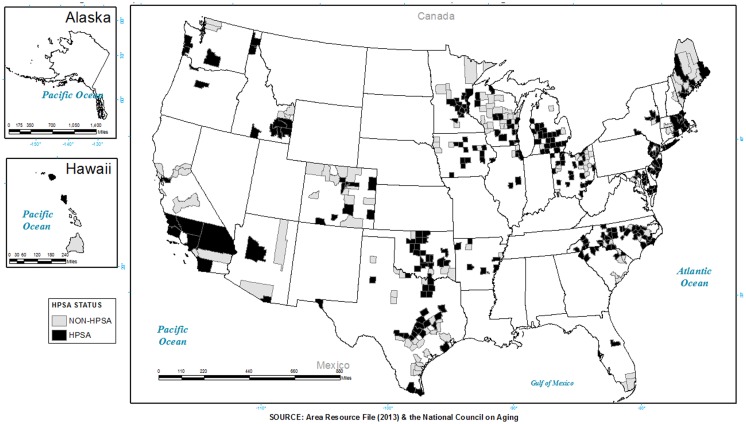
**Counties identified as a primary care health professional shortage area (HPSA) with a presence of evidence-based programs**.

Figure 2 shows where programs were delivered in areas classified as a HPSA. Gray shading represents where programs (i.e., AMOB/VLL, CDSMP, EF) were offered in a non-HPSA. Black shading represents where programs were offered in a HPSA (full or partial). As presented in the map (Figure 2), approximately 88.9% of the participants attended programs in a HPSA. A greater proportion of participants in a HPSA were served by EF at 92.9%, compared to 88.8% by CDSMP and 86.5% by AMOB/VLL. Again, programs were delivered in high need areas, but that the extent varied by state (also seen in Figure 1).

**Figure 3:**
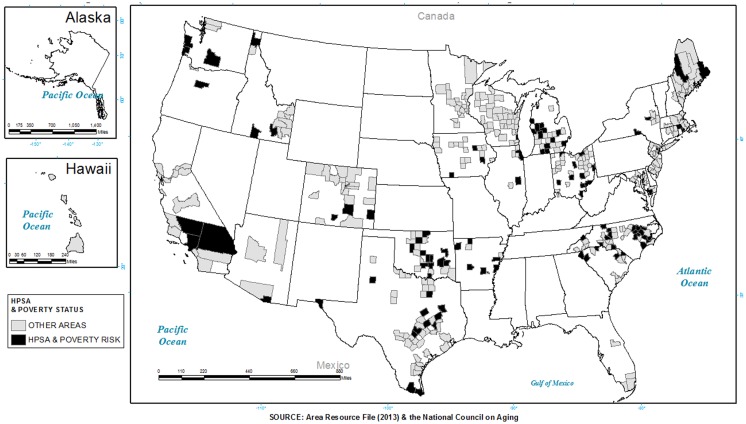
**Counties identified as a health professional shortage area, and having higher than the median poverty distribution with the presence of evidence-based programs**.

Figure 3 depicts the intersection of poverty and HPSA, where black shading represents places where programs were offered in areas classified as both high poverty (above the median percent poverty for 2008 measured at the county) and HPSA (full or partial). As seen, approximately 47.5% of the participants attended programs in areas with both higher poverty rates and that were a HPSA. A greater proportion of participants in these areas were served by EF at 55.7%, compared to 48.6% by AMOB/VLL and 44.3% by CDSMP.

### Availability of multiple evidence-based programs

Table 2 presents the distribution of counties that delivered one, two, and three of the EBP included in this study. Overall, 78.8% of counties labeled full-HPSA delivered only one EBP, 18.5% delivered two of the EBP, and 2.6% delivered all three EBP. Approximately 84% of rural counties delivered one of these EBP, and 1.6% delivered all three programs. Nearly 75% of counties within higher poverty areas delivered one EBP versus 2.3% that offered all three EBP.

**Table 2:** **Distribution of counties by availability of multiple evidence-based programs (CDSMP, AMOB/VLL, EF) by health professional shortage area (HPSA), rurality, and poverty status in 2008**.

	One program		Two programs		Three programs	
	*n*	%	*n*	%	*n*	%
**HPSA status**						
Full-HPSA	183^a^	78.9	43	18.5	6	2.6
Partial-HPSA	159	71.0	57^b^	25.5	8	3.6
Non-HPSA	135^a^	82.8	28^b^	17.2	0	0
**Rurality**						
Rural	216*	84.4	36*	14.1	4	1.6
Urban	261*	71.9	92	25.3	10	2.8
**Poverty rate**						
Above median	298*	75.6	85*	21.6	11*	2.8
At/below median	179*	79.6	43*	19.1	3*	1.3
Total	477	77.1	128	20.7	14	2.3

*Significantly (*p* < 0.05) different by characteristic (e.g., rurality).

*^a^Significantly (*p* < 0.05) different non-HPSA versus full-HPSA*.

*^b^Significantly (*p* < 0.05) different non-HPSA versus partial-HPSA*.

Table 3 presents the distribution of participants by counties that delivered one, two, and three of the EBP included in this study. Overall, 43.6% of participants attended programs in areas offering only one EBP, 39.6% attended programs offering two of the EBP, and 16.7% attended programs offering all three EBP. Fifty-nine percent of participants in rural counties had only one EBP available to them, and 12.5% had all three programs available in their counties. Approximately 13% of participants within higher poverty areas had all three EBP available in their counties versus 20.8% in areas with lower poverty rates. Among areas that were designated as a full-HPSA, the majority of participants were in areas where one or two EBP were available as compared to 6% in areas where all three EBP were available.

**Table 3:** **Distribution of participants by availability of multiple evidence-based programs (CDSMP, AMOB/VLL, EF) by health profes- sional shortage area (HPSA), rurality, and poverty status in 2008**.

	One program		Two programs		Three programs	
	*n*	%	*n*	%	*n*	%
**HPSA status**						
Full-HPSA	6,120^a^^,^^b^	43.9	6,976^a^^,^^b^	50.0	845^b^	6.1
Partial-HPSA	4,477^b^^,^^c^	34.7	4,208^b^^,^^c^	32.6	4,209^b^	32.6
Non-HPSA	2,577^a^^,^^c^	76.9	773^a^^,^^c^	23.1	0	0
**Rurality**						
Rural	3,128*	59.0	1,511*	28.5	662*	12.5
Urban	10,046*	40.4	10,446*	42.0	4,392*	17.7
**Poverty rate**						
Above median	5,946*	39.7	7,122*	47.6	1,894*	12.7
At/below median	7,228*	47.5	4,835*	31.8	3,160*	20.8
Total	13,174	43.6	11,957	39.6	5,054	16.7

*Significantly (*p* < 0.05) different by characteristic (e.g., rurality).

*^a^Significantly (*p* < 0.05) different non-HPSA versus full-HPSA*.

*^b^Significantly (*p* < 0.05) different non-HPSA versus partial-HPSA*.

*^c^Significantly (*p* < 0.05) different partial-HPSA versus full-HPSA*.

**Figure 4:**
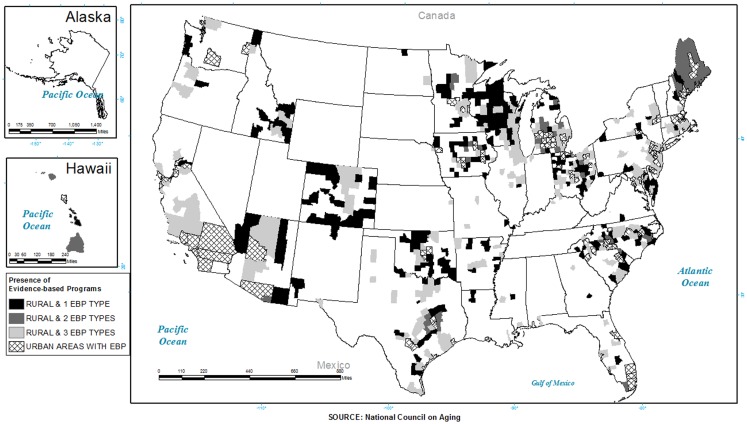
**Distribution of evidence-based programs (EnhanceFitness, A Matter of Balance/Volunteer Lay Leader Model and the Chronic Disease Self-Management Program) by county and rurality**.

Figure 4 shows the distribution of rural counties that delivered one versus two versus three of the EBP included in this study. As seen, there were very few areas that delivered all three programs (2.3%) and even fewer in rural counties (1.6%), and those that did (i.e., delivered all three programs) were concentrated in just a few states (e.g., AZ, CA, MA, NC, SC, TX).

Table 4 shows the distribution of counties by selected characteristics and programs. Overall, CDSMP was located in the largest number of counties at 419, followed by AMOB/VLL (253), and EF (103). In addition, the majority of counties offering EBP were located in a full or partial-HPSA (see Table 4). A higher proportion of the EBP were located in metropolitan areas, as compared to non-metropolitan areas. More counties offering these EBP were also located in lower poverty areas (compared to above the median poverty rate).

**Table 4:** **Distribution of counties by availability of evidence-based programs (CDSMP, AMOB/VLL, EF) by health professional shortage area (HPSA), rurality, and poverty status in 2008**.

	CDSMP		AMOB/VLL		EF		CDSMP and AMOB/VLL		CDSMP and EF		AMOB/VLL and EF	
	*n*	%	*n*	%	*n*	%	*n*	%	*n*	%	*n*	%
**HPSA status**												
Full-HPSA	165^a^	39.4	90	35.6	32^c^	31.1	37	34.9	12^a^	41.4	12^a^	34.3
Partial-HPSA	149^b^	35.6	97^b^	38.3	51^b^^,^ ^c^	49.5	45^b^	42.5	16^b^	55.2	20^b^	57.1
Non-HPSA	105^a^^,^^b^	25.1	66^b^	26.1	20^b^	19.4	24^b^	22.6	1^a^^,^^b^	3.5	3^a^^,^^b^	8.6
**Rurality**												
Rural	178*	42.5	81*	32.0	41*	39.8	27*	25.5	8*	27.6	13	37.1
Urban	241*	57.5	172*	68.0	62*	60.2	79*	74.5	21*	72.4	22	62.9
**Poverty rate**												
Above median	136*	32.5	93*	36.8	45	43.7	31*	29.3	7*	24.1	14	40.0
At/below median	283*	67.5	160*	63.2	58	56.3	75*	70.8	22*	75.9	21	60.0
Total	419		253		103		106		29		35	

*Significantly (*p* < 0.05) different by characteristic (e.g., rurality).

*^a^Significantly (*p* < 0.05) different non-HPSA versus full-HPSA*.

*^b^Significantly (*p* < 0.05) different non-HPSA versus partial-HPSA*.

*^c^Significantly (*p* < 0.05) different partial-HPSA versus full-HPSA*.

## Discussion

This study examines the delivery of three EBP delivered to vulnerable individuals (i.e., minority/ethnic individuals, those living in rural or HPSA areas, with low income, and those having one or more chronic conditions or advanced age) within 24 states through the 2006–2009 AoA Evidence-Based Disease and Disability Prevention Initiative and 3 states funded by the Atlantic Philanthropies. The findings reveal that the three EBP reached a substantial percentage of adults who were aged 75 years or older and had incomes below $15,000. The proportion of minority/ethnic participants in each of these three EBP was higher than the current proportion of minority/ethnic adults in US (approximately 22%) in 2012 (51). Additionally, among those with at least one chronic condition, the majority of these participants had comorbid conditions (i.e., two or more chronic conditions) overall and within each program. We note that an overwhelming number of women participated in these programs, which, in part, seems to reflect the larger proportion of women representing the American older adult population. However, this is frequently reported in other national studies of EBP for older adults (16–18, 20). The lower reach to males and ethnic minorities raises questions as to whether the programs lack saliency to specific subpopulations or whether the providers are finding it difficult to find the right strategies to recruit such subpopulations. Further research is needed to explore and examine ways in which nationally coordinated intervention efforts can recruit a greater proportion of diverse populations.

It is not surprising that CDSMP had the largest number of participants, given that all participating states were required to deliver this program, but could add other EBP desired by community partners. The overall distribution of programs (as seen in the figures) illustrates the limited reach within the 27 grantee states during this specific initiative. However, there has been subsequent growth in program dissemination and participant reach in recent years. For example, CDSMP was delivered in 27 funded states during the 2006–2009 initiative, but it was delivered in 45 states, the District of Columbia, and Puerto Rico, reaching more than 100,000 older adults from 2010 to 2012 (20). AMOB/VLL was offered in 24 states during 2006–2009, but is now available in over 30 states. Further, EF was delivered in 22 states and is now offered in over 25 states. We recognize that while more states are offering these EBP since the 2006–2009 initiative, there has been variability in their delivery, with some counties increasing their offerings, and others cutting back due to lack of funding.

Having multiple evidence-based interventions available to older adult populations provides an opportunity for better tailoring to the unique needs of seniors with a variety of chronic conditions. Such tailoring may be especially important for the most vulnerable participants (52, 53). Yet, the study data showed that the largest proportion of participants were located in areas where only one program type was offered, regardless of area characteristics. The data also showed that multiple programs are typically less likely to be offered in areas serving the most vulnerable populations (e.g., those living in low income or rural areas and in a HPSA). It was not surprising to find that these areas offered the least number of different programs, as this confirms prior research indicating rural residents have lower access to healthcare services than their urban counterparts (54–56) where there are typically fewer resources and greater distances to providers (57). Drawing from our collective experience implementing and disseminating EBP, we recommend some practical approaches for increasing the delivery of multiple programs in a given area. One approach may include building an infrastructure that can support multiple EBP (58). While the co-ordination of area agency on aging (AAA) funding varies by state (i.e., either centralized or decentralized infrastructure), these EBP may not be capable of reaching certain geographic locations. Moreover, even when communities want to offer these programs, they may not have the program delivery infrastructure to serve the demands in their communities. As such, more research is needed to better understand why states and AAA elect to offer only certain programs, as well as the infrastructure-related challenges associated with EBP delivery (especially as it pertains to multi-program implementation). Further, future research might explore why vulnerable adults only choose to participate in one program despite the potential benefits of participating in multiple programs. Another approach to enhance program delivery capacity could be offering cross-training opportunities for different lay leaders and healthcare professionals so they can lead workshops for multiple programs. Such an approach is being implemented by the Stanford Patient Education Research Center, which offers the suite of chronic disease self-management education programs (Retrieved from http://patienteducation.stanford.edu/training/). Another approach might be to address and solve transportation needs to and from sites offering programs that are often an issue in rural areas.

There were several limitations in the current study. First, this study only examined the three most prevalent EBP being delivered through the AoA Evidence-Based Disease and Disability Prevention Initiative and the Atlantic Philanthropies from 2006 to 2009. While these data are now over 5 years old, no other national database exists; hence, they are particularly powerful for illuminating the two research questions posed in this study. Second, only the three identified programs sponsored by this initiative were included, so that the study does not account for other EBP that might also have been offered by different sponsors. Third, the type of available data and amount of missing data is also a limitation to be acknowledged. In order to reach large numbers of participants being offered EBP through existing community organizations, the amount of required data for this study was limited to a few basic demographic and programmatic factors. Even with this streamlined data collection protocol, there was substantial missing data due to the inability of community providers to systematically collect and release all requested data (e.g., in some healthcare systems providers were not able to release information due to institutional review board restrictions). However, large amounts of missing administrative or programmatic data are not uncommon in evidence-based community interventions (59–62). In addition, analyses that include chronic conditions were limited to data for individuals with one or more chronic conditions. Cases reporting no chronic conditions were omitted because it was impossible to determine whether these cases had no chronic conditions or neglected to respond to these survey items (i.e., missing data). Our analyses do not take into consideration the level of social support among participants; however, future analyses should include this as a possible factor associated with participant outcomes.

Finally, we could not measure the actual penetration among all *possible* participants for these EBP. Future research should examine the extent of reach among those potential participants for these EBP. The cross-sectional nature of the study prevents analysis of trends over time; however, the goal of this study was to measure the overall reach among vulnerable adults, and service delivery characteristics during the initiative period. Future studies should also identify strategies for identifying the dissemination of multiple EBP throughout the US and their interactive impacts on our aging population. At the current time, there is no mechanism for doing so. However, we should look toward a national inventory of EBP for seniors, potentially linked to healthcare utilization outcomes, or community assessments that can track county level changes in health and functioning.

Study findings demonstrate that individually these three EBP have the capacity to appeal to vulnerable populations. Going forward, the challenge is to create an efficient national infrastructure that encourages widespread adoption and bundling of these programs for delivery in underserved populations and areas. Systematical engagement and meaningful involvement of vulnerable populations to fine tune outreach strategies, enhancing linkages with the healthcare system that includes advocating for the importance of evidence-based programing, building marketing strategies and business models, and accelerating adaptation of evidence-based programing are approaches that program administrators, policy makers, and funders can use to continue outreach to vulnerable older adults (63).

New federal initiatives (e.g., Affordable Care Act) (62) are encouraging the aging services network sector to work collaboratively with public health and medical care sectors and other key stakeholders responsible for improving the health and functioning of our rapidly escalating population of older adults with multiple chronic conditions. Growing and sustaining EBP in a diversity of delivery sites that attract a broader range of participants will be critical for achieving a greater population health impact (16).

## Conflict of Interest Statement

Samuel D. Towne Jr. has no conflicts of interest to disclose. Neither himself nor this institution at any time received payment or services from a third party for any aspect of the submitted work. Dr. Towne has no financial relationships with entities that could be perceived to influence, or that give the appearance of potentially influencing, what he wrote in the submitted work. Dr. Towne has no patents or copyrights to declare (whether pending, issued, licensed and/or receiving royalties) relevant to the work. Dr. Towne has no other relationships or activities that readers could perceive to have influenced, or that give the appearance of potentially influencing, what he wrote in the submitted work. Matthew Lee Smith, PhD, MPH, CHES has no conflicts of interest to disclose. Neither myself nor this institution at any time received payment or services from a third party for any aspect of the submitted work. Dr. Smith has no financial relationships with entities that could be perceived to influence, or that give the appearance of potentially influencing, what he wrote in the submitted work. Dr. Smith has no patents or copyrights to declare (whether pending, issued, licensed, and/or receiving royalties) relevant to the work. Dr. Smith has no other relationships or activities that readers could perceive to have influenced, or that give the appearance of potentially influencing, what he wrote in the submitted work. SangNam Ahn, PhD, MPSA has no conflicts of interest to disclose. Basia Belza, PhD, RN, FAAN has no conflicts of interests and he or his institution did not receive payment or services from a third party. Mary Altpeter, PhD, MSW, MPA has no conflicts of interest to disclose. Kristie Patton Kulinski, MSW has no conflicts of interest to disclose. Marcia G. Ory, PhD, MPH has no conflicts of interest to disclose.

This paper is included in the Research Topic, “Evidence-Based Programming for Older Adults.” This Research Topic received partial funding from multiple government and private organizations/agencies; however, the views, findings, and conclusions in these articles are those of the authors and do not necessarily represent the official position of these organizations/agencies. All papers published in the Research Topic received peer review from members of the Frontiers in Public Health (Public Health Education and Promotion section) panel of Review Editors. Because this Research Topic represents work closely associated with a nationwide evidence-based movement in the US, many of the authors and/or Review Editors may have worked together previously in some fashion. Review Editors were purposively selected based on their expertise with evaluation and/or evidence-based programming for older adults. Review Editors were independent of named authors on any given article published in this volume.
